# Functional Amyloid and Other Protein Fibers in the Biofilm Matrix^☆^

**DOI:** 10.1016/j.jmb.2018.07.026

**Published:** 2018-10-12

**Authors:** Elliot Erskine, Cait E. MacPhee, Nicola R. Stanley-Wall

**Affiliations:** 1 Division of Molecular Microbiology, School of Life Sciences, University of Dundee, Dundee DD1 5EH, UK; 2 James Clerk Maxwell Building, School of Physics, University of Edinburgh, The Kings Buildings, Mayfield Road, Edinburgh, EH9 3JZ, UK

**Keywords:** functional amyloid fibers, biofilm matrix, curli, TasA, PSM, eDNA, extracellular DNA, ThT, thioflavin T, CD, circular dichroism, TEM, transmission electron microscopy, MTP, *M. tuberculosis* pili, HR, hypersensitivity response

## Abstract

Biofilms are ubiquitous in the natural and man-made environment. They are defined as microbes that are encapsulated in an extracellular, self-produced, biofilm matrix. Growing evidence from the genetic and biochemical analysis of single species biofilms has linked the presence of fibrous proteins to a functional biofilm matrix. Some of these fibers have been described as functional amyloid or amyloid-like fibers. Here we provide an overview of the biophysical and biological data for a wide range of protein fibers found in the biofilm matrix of Gram-positive and Gram-negative bacteria.

## Highlights

• Biofilms are complex communities of bacteria encased in an extracellular matrix.
• The biofilm matrix frequently contains fibrous proteins.
• Many of the proteins have been designated amyloid-like fibers.
• We review the designation in light of biochemical and biophysical data.

## Graphical abstract

**Figure:**
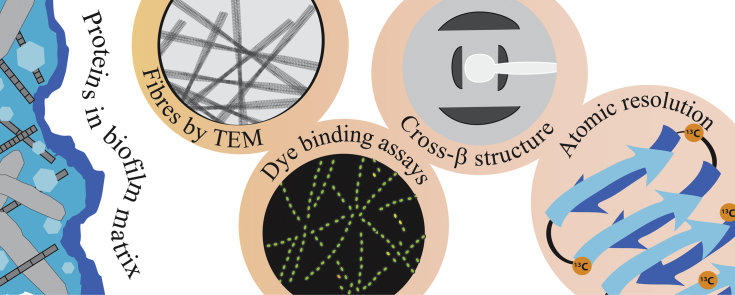

## Biofilms and Fiber-forming Proteins of the Matrix

Biofilms are communities of microorganisms attached to a surface and encompassed by a self-produced extracellular matrix. The biofilm matrix is dynamic and fulfils multiple functions including nutrient sequestration and water adsorption, shielding the resident cells from environmental stress and competition [1], and acting as a signaling facilitator for cells both within and outside the biofilm [2]. There is great diversity in composition of the biofilm matrix across polymicrobial and between single-species biofilms; however, commonly occurring constituent parts include polysaccharides, extracellular DNA (eDNA), lipids and proteins, some of which are fibrous in nature [1], [3]. Growing evidence suggests that fiber-forming proteins provide structural integrity to many biofilms [4], [5], [6], which ultimately provides protection for the bacteria, for example, from phage predation [7].

Amyloid fibers have serious negative medical implications and are predominately associated with neuropathic, single-organ and systemic diseases that are characterized by extracellular insoluble protein deposition [8]. In contrast with these disease-associated fibers, an emerging field of study concerns the “functional” amyloid fold, with proposed examples occurring in mammals, fungi and bacteria [9], [10], [11]. Many of the fiber-forming proteins in the biofilm matrix have been described as “amyloid” or “amyloid-like.” Here, however, we posit that, in the context of the biofilm matrix, the term “functional amyloid fiber” has been broadened to include a diverse range of protein structures. For example, the term amyloid-fiber has been associated with both β-helix [12], [13] and α-helical fiber structures [14]. We overview the historical significance of the term “amyloid” and its medical origins and significance. We then describe the structure and function of fibers that have been previously identified as “amyloid-like” in biofilms from a broad range of species.

## Amyloid Fibers in Disease

Mammalian systems depend on complex regulatory networks to deal with misfolded proteins appropriately, and dysregulation of these processes can cause disease. When a native protein misfolds and deposits as insoluble fibers with a cross-β structure, the resulting disease is termed an amyloidosis [8]. The site of deposition can be in single organs, such as the pancreas in type 2 diabetes [15], in multiple organs in systemic amyloidosis, or neuropathic as in Alzheimer's and Parkinson's disease [16]. In all of these diseases, the insoluble structure of the amyloid fiber has devastating consequences on homeostasis.

This same insolubility long hindered structural elucidation of the amyloid fiber, but awareness of the extracellular deposits by histopathology can be traced back to the 18th century [17]. Accordingly, medical notes are full of archaic descriptors like “lardaceous,” which stemmed from the waxy deposits in diseased kidneys, spleens and livers resembling bacon in the eyes of French pathologists [17]. The name “amyloid” was introduced in 1854 by Virchow, due to the blue-black color change of bound iodine in tissue samples from the brain [18]. During the mid-20th century, the publication of an amyloid fiber extraction technique (the Pras method [19]) allowed the molecular structure of amyloid fibers to be probed *in vitro*. This involved homogenizing amyloid-laden tissue and removing the soluble fraction of protein, and then isolating the insoluble amyloid-containing fraction by repeated centrifugation. This also removes any endogenous fiber-associated factors and possibly alters the structure of the amyloid fiber in the process. In the next section, we overview the methods used to analyze fiber-forming proteins.

## Methodologies for Understanding the Amyloid Fiber

### Physical characteristics of amyloid fibers

**Fig. 1:**
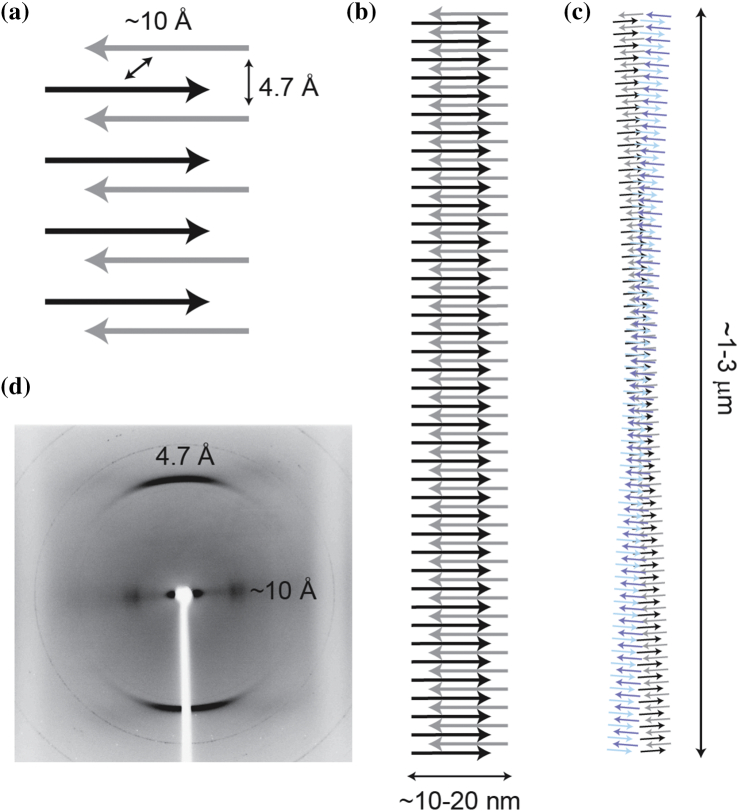
Characteristic structure of an amyloid-like fibril. In amyloid-like fibers, proteins adopt a predominantly β-sheet conformation (a). β-Strands are arranged orthogonally to the fiber axis, held together by interactions between sidechains in the fiber core, and by an extensive network of hydrogen bonds running parallel to the fiber axis (b, c). The distance between β-strands parallel to the fiber axis is a characteristic 4.7 Å, reflecting the hydrogen bond array, and the intersheet distance ranges between 6 and 12 Å. These distances, and therefore this “cross-β” arrangement, are confirmed by X-ray fiber diffraction, which reveals two orthogonal reflections (d). Reprinted with permission from Ref. [20]. Copyright 2008 American Chemical Society.

Amyloid fibers are insoluble in SDS and are resistant to proteolytic cleavage. This is due to the “cross-β” structure common to all amyloid fibers where β-sheets run parallel to the fiber axis composed of β-strands stacked vertically, like the rungs of a ladder (Fig. 1a). The β-sheets, which are typically in-register, are close enough to be governed by Van der Waals forces, and in some cases [21], [22], the sidechains interdigitate to form a “steric zipper,” thereby excluding water from the interior of the protofilament. The individual β-strands of the protofilament can be arranged in a parallel or antiparallel fashion, each extensively hydrogen-bonded *via* amide and carboxyl groups in the backbone of the polypeptide chain. These protofilaments stack to give the quaternary structure. It is this combination of Van der Waals interactions and an extensive hydrogen-bonded network that gives amyloid fibers their extraordinary stability.

### Direct dyes

In the early 20th century, it was discovered that the extracellular deposits bound the dye Congo red and exhibited birefringence: the bound dye emits a range of colors, typically “apple-green,” under crossed polarizers [23]. The common pitfalls that should be considered when using Congo red are extensively reviewed elsewhere [24], but crucially, this dye binds many polymeric substances including DNA and cellulose. It is common to see Congo red used in conjunction with another dye, thioflavin T (ThT). First introduced in 1959, ThT has long been used to diagnose amyloidosis [25] and to follow the kinetics of amyloid polymerization *in vitro* [26], but like Congo red, ThT has been demonstrated to exhibit false-positive binding to other polymers, and also proteins and protein aggregates that are not in the amyloid conformation [27], [28], [29]. The exact molecular mechanism that underpins ThT binding is still being investigated, but current consensus holds that the increase in fluorescence intensity is dependent on the restriction of the internal molecular rotation when the dye monomer is bound, or as viscosity of the microenvironment increases [30], [31].

### Immunolabeling

An additional technique used to identify amyloid species is immunolabeling. There are antibodies available that have been differentially raised against fibrillar (e.g., WO1/WO2 [32]) and soluble oligomeric amyloid species (e.g., A11 [33]). Despite showing sensitivity toward the fibrillar and oligomeric amyloid fold, to the best of our knowledge, there has been no systematic testing of the specificity of these antibodies for false-positive cross-reactivity with other bacterial filamentous structures such as flagella or with other β-rich structures, for example, β-solenoid domains.

### Structural characterization

#### Circular dichroism

Circular dichroism (CD) spectroscopy measures the difference in absorption of right and left polarized light by chiral molecules. The CD spectra at far-UV wavelength of proteins are influenced by the three-dimensional structure of the amide backbone, rather than the individual chiral amino acids, and therefore can be used to investigate polypetide secondary structure [34]. In the far-UV spectrum, the ratio of different secondary structural features can be identified; for amyloid structures, this is predominately β-sheet (Fig. 1a). When first introduced, it was thought that the technique could not provide adequate information on the arrangement of β-sheet secondary structure elements, but advancements have been made in the field [35].

#### X-ray diffraction

Additional structural information can be obtained by analyzing the molecular arrangement of the protofilament (Fig. 1b). Through observing the diffraction pattern of a beam of electrons (giving small angle patterning) or more commonly X-rays (allowing a wider range) through the insoluble fiber, it is possible to resolve the tertiary structure [36]. To perform this analysis, a droplet of a protein sample is suspended on a stretch frame apparatus and allowed to dry. As it dries, proteins fibrils (Fig. 1c) within the sample ideally become aligned. Since, in the case of amyloid fibers, the cross-β structure of the fiber is constant along the fiber axis, the patterning shows two reflections, characteristic of the inter-strand (4.7 Å) and inter-sheet (6–12 Å) distances. Thus, the term “cross-β” arises from the fact that these two reflections are orthogonal: the inter-strand distance is seen on the meridian, and the inter-sheet distance on the equator (Fig. 1d).

#### Atomic resolution

More recent technological advancements have enabled the atomic structure of fibers to be elucidated, albeit in a relatively limited number of cases (reviewed in Ref. [37]). Through a combination of solid-state nuclear magnetic resonance and mass/length estimates, the structure of protofilaments assembled from the Aβ peptide and a transthyretin peptide have been mapped [38], and cryo-electron microscopy has been used to determine the structure of native tau filaments extracted from a patient with Alzheimer's disease [39]. Interestingly, the tau fibers form an elaborate mixed architecture comprising two canonical cross-β structures, connected by a β-helix motif. Other atomic resolution studies include that of the Het-S fungal prion-forming domain, where the dry interface has three sides in a triangular β-solenoid [40]. The use of small internal peptides derived from full-length proteins as model systems for amyloid fiber formation is further complicated by the possibility of conformational variants. For example, for the hexapeptide “GNNQQNY” derived from the N-terminal region of the yeast prion protein Sup35, different conformations of the cross-β backbone between microcrystalline and fibrous assemblies have been detected by magic angle spinning solid-state NMR [41]. Whether this represents true heterogeneity in the cross-β motif is debatable; small-angle X-ray scattering studies show a single backbone conformation [42]. Furthermore, variation between the hexapeptide and the full-length Sup35 assembly has been noted, the latter reported to form amyloid fibers of out-of-register parallel structure [43]. Overall, the diversity that has emerged from atomic resolution studies has presented many questions regarding sample preparation and whether legitimate comparison can be made between *in silico, in* *vitro*, and *in vivo* derived structures.

#### Considerations

As the structural investigations have progressed, so has the understanding of the mechanisms that result in globular, folded proteins adopting the misfolded cross-β structure. By introducing environmental stress in the form of temperature or pH changes, it is possible to induce the amyloid fiber fold in any polypeptide chain [44], [45]; even those consisting of a single repeating amino acid. These are biophysically indistinguishable from the disease-causing amyloid fibers [44]. This is an important consideration when extracting novel proteins, ensuring that neutral conditions are used so as not to influence the energy landscape of the folded protein, which might otherwise bias toward inadvertent protein self-assembly.

## Protein Fibers in Biofilms

A survey investigating the abundance of amyloid fibers in natural biofilms was undertaken in sewage filtration plants and by using the amyloid fiber WO1 antibody, coupled with phyla-specific oligonucleotide probe-based fluorescence *in situ* hybridization revealed that 10%–40% of bacteria produced elements that bound to the WO1 antibody [46]. However, the identification of a novel functional fiber protein typically begins with an initial observation of an extracellular fiber network by electron microscopy. This is then followed by either native extraction or production of the recombinant protein in another species, commonly *Escherichia coli,* to allow a closer examination of its biophysical properties. If the protein proves to be insoluble in common solvents and therefore cannot be analyzed by SDS-PAGE, it may be necessary to expose it to harsh conditions. However, it is worth noting that this treatment alone may induce amyloid fiber conversion of the target protein regardless of prior conformation [44]. After extraction of the protein fibers, subsequent structural analysis needs to be undertaken to ascertain the molecular structure of the protein and to ultimately link this with its role in the biofilm matrix.

In the following sections, we will examine matrix-associated fibers that have been classified as amyloid-like, highlighting the variety of fiber structures and functions. We collate the properties of these fibers that have been collected through biophysical means and through associated traits such as resistance to SDS and susceptibility to proteolytic cleavage (Table 1).

**Table 1:** Biochemical and biophysical properties of bacteria protein fibers

Species	Protein^a^	Physiological function^b^	Source	Birefringence	Congo red	ThT	Antibodies	Secondary structure (XRD, NMR)	Secondary structure (CD, FTIR)	Ultrastructure (EM)	Soluble in SDS	Protease resistant	Isolated fiber biological activity
*E. coli* and *S.typhimurium*	Curli/Tafi [47]	Structural biofilm matrix molecule [47]	Native, extracted by sequential centrifugation	Green/yellow [48]	Red shift [49]	Enhanced fluorescence [49]	No data	No data	β-sheet [49], [50]	fiber network [47]	No [51]	Yes [51]	Genetic complementation [52]
			CsgA Full-length recombinant His-tagged	No data	Red shift [49]	Enhanced fluorescence [53]	A11 + ve intermediates [53]	Not in-register; diffraction rings at 4.8 Å, 9 Å [12]	β-sheet [49]	Needle-like fibers [49]	No [49]	Yes [12]	Polymerized by *csgA* − cells [52]
			CsgB Recombinant Δ18 aa from C-term	No data	Red shift	Enhanced fluorescence [54]	No data	No data	β-sheet [54]	Needle-like fibers [54]	No [54]	No Data	Can nucleate CsgA *in vitro*, mislocalizes *in vivo*[54]
			CsgB full-length recombinant	No data	No data	Enhanced fluorescence [12]	No data	Not in-register; diffraction rings at 4.7 Å, 9 Å [12]	Mixed β-sheet/β-turn/α-helical [12]	Fibers [12]	No [12]	No data	No data
*Pseudomonas* sp.	Fap [55]	Structural biofilm matrix molecule [55]	Native, extracted by boiling in SDS	Data not shown [55]	No data	Enhanced fluorescence [55]	WO1 + ve fibers [56]	No data	β-Sheet post-formic acid [55]	Fibers [55]	No [55]	No data	No data
			Whole fapA-H operon Recombinant	No data	No data	No data	No data	Diffraction rings at 4.15 Å, 4.34 Å, 4.61 Å, 6.32 Å, 11.53 Å [55]	β-Sheet [55]	Fibers [55]	No [57]	No data	Increased aggregation *in vivo*[57]
*Bacillus* sp.	TasA [58]	Structural biofilm matrix molecule [59]	Native extracted by salting	No data	No red shift [59]	Enhanced fluorescence [59]	A11 + ve intermediates [59]	No data	α-Helical [59]	Extracellular fibers [59]	Yes [59]	No data	Yes [59]
			Recombinant Δ22 aa from C-term	No data	No data	No data	No data	Jellyroll [60]	No data	No data	No data	No data	N/A
*M. tuberculosis*	MTP [61]	Structural biofilm matrix molecule [61]	Native	No data	Data not shown [61]	No data	No data	No data	No data	Extracellular fibers [61]; Disputed [62]	Yes [61]	No data	Yes [63]; Disputed [62]
*S. mutans*	P1 [64]	Structural biofilm matrix molecule	His-tagged Recombinant P1 (stirred)	Green/yellow [48]	No red shift [48]	Enhanced fluorescence [48]	No data	No data	No data	Needle-like fibers [48]	No data	No data	No data
	WapA (Antigen A)	Cell-wall associated	Produced by curli system recombinant	Green/Yellow [65]	No data	Enhanced fluorescence [65]	No data	No data	α-Helical to β-sheet at pH 4 [65]	Dark extracellular aggregates [65]	Yes [65]	No data	No data
	SMU_63c [65]	Putative competence involvement [65]	Produced by curli system recombinant	Green/Yellow [65]	No data	Enhanced fluorescence [65]	No data	No data	α-Helical to β-sheet at pH 4 [65]	Dark extracellular aggregates [65]	Yes [65]	No data	No data
*Staphylococcus* species	Phenol-soluble modulins [66]	Putative biofilm matrix molecule [67]	Native	No data	Red shift by synthetic internal peptide [67]	Enhanced fluorescence by internal peptide [67]	No data	No data	β-Sheet [67]	Extracellular fibers [67]; Disputed [68]	No [67]	Yes [67]	Biofilm [67]; Disputed [68]
		PSMα3 [14]	Synthetic PDB:5155	No data	No data	Enhanced fluorescence [14]	No data	Diffraction rings at 32 Å, 11.5 Å, 9.6 Å, 4.5 Å [14]	Fiber and monomer α-helical [14]	Twisted bundles of fibers [14]	No data	No data	Cytotoxicity [14]
	SuhB	Putative biofilm matrix molecule [69]	His-tagged recombinant	No data	Red shift [70]	Enhanced fluorescence [70]	No data	Diffraction rings at 3.8 Å, 4.7 Å, 10–11 Å [70]	β-Sheet [70]	Sheet-like fiber network [70]	Yes [70]	No data	No data
	Bap	Adhesion during biofilm formation [71]	Recombinant Internal peptide	No data	Red shift at pH 4.5 [71]	Enhanced fluorescence at pH 4.5 [71]	No data	No data	β-Sheet; 205 nm minima of internal peptide [71]	Compares peptides to native by TEM [71]	No [71]	No [71]	Cell clumping [71]
*Gallibacterium anatis*	Tu Elongation factor	Adhesion during biofilm formation [72]	His-tagged recombinant	No data	No data	No data	No data	No data	No data	Dark extracellular aggregates [72]	Yes [72]	No data	No data
*Xanthomonas anxidopidos*	HpaG [73]	Plant pathogenicity [73]	His tagged Recombinant	Green/yellow [74]	Red shift [74]	No data	No data	No data	α-Helical to β-sheet [74]	Network of fibers [74]	Yes [74]	Limited resistance [74]	No data
*Legionella pneumophila*	No data	Biofilm-associated structural [75]	Native	Red binding [75]	No data	Enhanced fluorescence [75]	WO2 + ve fibers [75]	No data	No data	No data	No data	No data	No data
*Solibacillus silvestris* AM1	BE-AM1 [76]	Bioemulsfier [76]	Native	Green/Yellow [76]	No data	No data	No data	No data	β-Sheet; Mixed α-helical and β-sheet [76]	[76]	No data	No data	No data
*Klebsiella pneumoniae*	Microcin E492 [77]	Modulation of cytotoxic active monomer [78]	MccE492 full-length recombinant with immunity determinant	No data	Red-shift [78]	Enhanced fluorescence [78]	A11 + ve intermediates [79]	Diffraction rings at 4.71 Å, 9.92 Å [80]	β-Sheet [78]	Fibers polymerize after 220 rpm shaking 15 h [78]	Yes [78]	No [78]	No data
*Streptomyces coelicolor*	Rodlins [81]; Chaplins [82]	Formation of rodlet layer [83]	ChpA-F Native extracts	No data	No data	Enhanced fluorescence [84]	No data	No data	Random coil to β-sheet [84]	4- to 6-nm wide lines shadowing on formvar grids [84]	No [84]	No data	Aerial hyphae formation [84]
			Synthetic ChpA-F peptides	No data	No data	Enhanced fluorescence [85]	No data	Diffraction rings at 4.7 Å and 10 Å [85]	β-Sheet [85]	Fibers [85]	No data	No data	Aerial hyphae formation [85]
			His-tagged RdlB recombinant	No data	No data	Enhanced fluorescence [86]	No data	Diffraction rings at 4.7 Å, 10.2 Å and 30.9 Å [86]	Random coil to β-sheet [86]	Twisted bundles of fibers [86]	No data	No data	Aerial hyphae formation [86]

a The reference indicated is the first identification of the protein.

b Physiological function assigned.

## Amyloid-like Curli Fibers of *E. coli* and Related Species

**Fig. 2:**
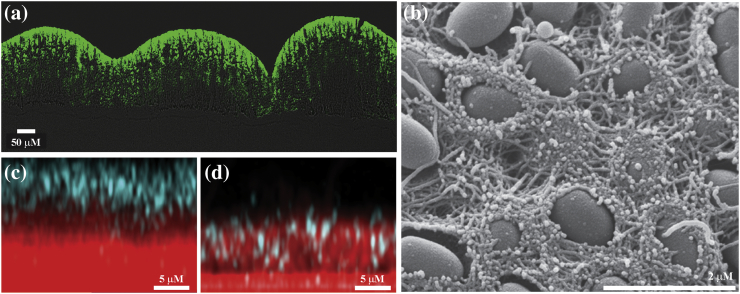
A structural and protective role for curli in the *E. coli* biofilm matrix. (a) The localization of the curli in a vertical cross section of a 7-day-old *E. coli* biofilm stained with thioflavin S (ThS). Merged bright-field and false-colored fluorescence for ThS. (b) Scanning electron micrograph in high vacuum mode of the top view of a 7-day-old K-12 W3110 cellulose-free *E. coli* biofilm. The cells are round and the curli baskets are visible. (c, d) Maximum intensity z-projections of phage localization (cyan) after 8-h exposure of 72 h *E. coli* wild-type (c) and curli-deficient (d) biofilm. Cells are in red. Parts a and b [89] and c and d [7] are reproduced with permission along with the corresponding legend.

The foremost studied biofilm-associated functional amyloid-like fibers are the curli fibers from *Enterobacteriaceae*. Highly conserved between the Gram-negative *E. coli* and *Salmonella* species, curli extracellular fiber networks were first identified by transmission electron microscopy (TEM) and noted for their insolubility [47], [51]. The *r*ed, *d*ry *a*nd *r*ough (rdar) colony morphology observed on agar plates supplemented with Congo red dye is curli dependant in both species [87]. The red coloration provided a simple way of screening for mutations in associated genes, leading to the mapping of the large curli-related gene network [88]. Curli expression is dependent on the starvation response resulting from stratification of the biofilm and thus is more prominent where there are non-dividing cells furthest from nutrients (Fig. 2a) [89]. The cells in this zone become curliated and form a network of “cell-moulded baskets” throughout the intercellular space providing essential structure to the colony [89] (Fig. 2B). Production of curli is also critical for initial adhesion to both biotic and abiotic surfaces and is linked to environmental resistance and pathogenesis [90], [91], [92], [93]. Most recently, in addition to aiding cell adhesion, curli have been shown to both sequester and hinder diffusion of a predatory phage, preventing it from reaching the interior of the biofilm community [7] (Fig. 2c, d), directly demonstrating the protection that is provided to the cells residing in a biofilm. In addition to the curli, some strains of *E. coli* also (or only in some cases) produce the polysaccharide cellulose carrying a phosphoethanolamine modification [94], which together with the protein fibers produce a nanocomposite matrix material [95].

Curli have a dedicated secretion system, chaperone proteins and inhibitors, all encoded by the *csgBAC* and *csgDEFG* operons, with regulation and polymerization being tightly controlled [96], [97]. CsgA is the major protein subunit of curli; the encoding gene *csgA* is co-transcribed with *csgB,* and both gene products are translocated across the inner membrane by the Sec pathway and outer membrane by the CsgGFE secretion complex [21], [98]. The current model proposed has CsgB embedded in the outer membrane where it acts as a nucleator for the polymerization of the CsgA unstructured monomers into the fiber (along with a minority of CsgB) [99], [100]. When extracted by sequential differential centrifugation, the native fibers were found to be insoluble in SDS and had binding affinity for the dyes Congo red and ThT [49]. Analysis of the curli fibers by CD revealed a single minimum at 218 nm, typical of β-sheet structure. In combination, this led to the categorization of curli as amyloid fibers [49]. Recombinant CsgA and CsgB spontaneously polymerize, and it is these *in vitro* fibers that have been used for structural characterization by solid-state NMR and X-ray diffraction [12], [53]. Structural analysis of recombinant CsgA fibers by solid state-NMR revealed interatomic distances consistent with a β-helix architecture, but the data are not yet sufficient to rule out other architectures [12]. X-ray fiber diffraction [50] suggests a structure consistent with a β-arrangement, but either the recombinant fibrils were not sufficiently aligned within the fiber to clearly demonstrate the cross-β architecture, or an alternative β-structure is adopted. The relevance of these studies to the *in vivo* environment is unclear as it is possible there are modifications to the atomic structure that are driven by the nucleating role of CsgB [50]. Despite *in vivo* production of curli being linked to both the rdar colony morphotype and adhesion of the biofilm to abiotic and biotic surfaces, there is currently no direct evidence for functional activity of the recombinant CsgA or CsgB fibers assembled *in vitro*. The closest available data for functionality of recombinant CsgA *in vivo* are the nucleation of purified CsgA monomers at the cell surface of a *csgA* mutant, likely to be mediated by CsgB at the membrane [52].

## The Fap Fibers, an Emerging Story in *Pseudomonas* Species

Biofilm formation represents a survival strategy of *Pseudomonas aeruginosa* in the lung environment, as well as in wounds and on catheters [101]. The biofilm matrix components have been well characterized and include the capsule and aggregative polysaccharides, eDNA and appendages such as type IV pili and flagella (these matrix components have been reviewed extensively elsewhere [102]). In addition, there are the Fap fibers (that comprised the FapC subunit) that were initially identified in the *Pseudomonas fluorescens* biofilm matrix [55]. Evidence for the Fap fiber being functional comes from complementation of a Δ*fapABCDEF* strain with the complete operon encoded on a plasmid leading to increased aggregation compared with wild-type [103]. Moreover, recombinant expression of the *fap* operon is correlated with increased aggregation in *P. aeruginosa, P. putida, P. fluorescens* and *E. coli* [55], [57]. Native extracts of the fibers were described as having similar amyloid-like properties to the curli, displaying positive Congo red and ThT binding, coupled with insolubility in SDS and resistance to proteinase-K degradation [55]. Solution CD spectra collected from natively extracted Fap fibers reveals a 217-nm minima indicative of β-sheet secondary structure [55]. Similar characteristics were seen for recombinant FapC, and additional X-ray diffraction analysis of the fibers structure gave a range of reflections: 4.15, 4.34, 4.61, 6.32 and 11.53 Å [55]. The fibrils within the fiber employed for X-ray diffraction were not sufficiently aligned to provide definitive evidence for a cross-β arrangement.

## Phenol-Soluble Modulins by *Staphylococci*—A “Cross-α” Helix

*Staphylococcus epidermidis* and *Staphylococcus aureus* are the predominant colonizers of medical implants in the hospital setting and are often identified as opportunistic wound pathogens [104]. When examining staphylococci biofilms, the matrix composition is diverse and can be dependent or independent of polysaccharide internal adhesins [104]. Polysaccharide internal adhesins-independent biofilms rely on proteinaceous components and are especially prevalent in *S. epidermidis* infections [105]. The phenol-soluble modulins (PSMα1–4, PSMβ1–2 and γ-toxin) were first identified as a complex secreted by *S. epidermidis* acting as cytotoxic virulence factors involved in cytotoxicity and immunomodulation [66]. Investigations of the role played by these inflammatory proteins during biofilm formation began when fibrous material extracted from *S. aureus* was identified to contain αPSM and γPSM protein by mass spectrometric analysis [67]. Subsequent structural analysis was performed on synthetic αPSM1–4, βPSM1–2 and γ-toxin, which spontaneously assembled [67]. The CD spectra collected from each of the synthetic PSM peptides displayed β-sheet secondary structural features [67]. A role for eDNA in triggering PSM fiber polymerization has been explored [106], but it is unclear if formation of these fibers is biologically relevant, and an alternative hypothesis has been postulated where the PSM form a protective barrier on the eDNA [68]. Evidence that the synthetic αPSM3 fibers are not amyloid-like fibers was derived from X-ray diffraction of a microcrystalline form, where a “cross-α” structure was revealed [14]. Despite adhering to a general patterning reminiscent of the cross-β hallmark, this protein has a very different structure and can be considered a novel fiber form in bacteria if it is found natively in the biofilm. Recent proteomic analysis of an *S. aureus in vivo* infection model did not identify any PSM in the implant-associated biofilm matrix [107], highlighting the variation that can be generated when comparing *in vitro* and *in vivo* models.

## Non-amyloidogenic TasA Fibers Formed by *Bacillus subtilis*

*Bacillus subtilis* is a Gram-positive, spore-forming bacteria found ubiquitously in the soil and is a model organism for collective behaviors and differentiation [108], [109]. *B. subtilis* (strain NCIB3610) forms rugose biofilm colonies on agar and floating pellicles at air-liquid interfaces that are characterized by a dependency on matrix production [110]. The biofilm matrix contains a fiber-forming protein TasA that is essential for biofilm rugosity and is encoded as part of a three-gene operon: *tapA–sipW–tasA* [59], [111]. TapA has been reported as an accessory protein needed for TasA function, possibly as a nucleating species on the cell surface [112], and SipW is a dedicated signal peptidase, responsible for processing both the TapA and TasA proteins [58].

Structural and biochemical analysis of TasA has been performed using protein extracted from *B. subtilis* [59], [113] and with recombinant protein generated in *E. coli* [60]. Initial experiments were performed using TasA oligomers extracted from an exopolysaccharide negative, matrix-enriched *B. subtilis* strain [59], [113]. Here, solution CD spectra identified α-helical elements and a sensitivity to SDS depolymerization was observed [59], [113]. There was no red shift in Congo red absorbance, but the extracted protein did enhance the fluorescence of ThT [59]. Structural analysis of a truncated recombinant TasA monomer (amino acids 28–239 of the 261-amino-acid protein) by X-ray crystallography revealed that two antiparallel β-sheets are flanked by helices and loop regions (also known as a jellyroll conformation) [60]. Crucially, structural evidence of amyloid-like fibers is absent [59], [60] until the protein is exposed to acidic pH when the β-content increases [59], and cross-β X-ray diffraction patterns are recorded [60]. Although both natively extracted [59] and recombinant fibers [60] have been shown to be biologically active, the biological activity of the acid-treated form has not been shown. Finally, the requirement for TapA in the formation of TasA fibers that are functional *in vivo* has been refuted [114]. The significance of the mischaracterization of functional fibers as amyloid-like should not be understated, as TasA has been used in anti-amyloid drug screens [115], [116]. *In toto*, while the ability of TasA to form functional fibers associated with the biofilm matrix is not in doubt, these fibers are not amyloid-like in nature.

## *Mycobacterium tuberculosis* Pili

Biofilm formation by mycobacteria has been suggested to play a role in the environmental persistence and pathogenesis of non-tuberculous *Mycobacterium* opportunistic pathogens [117]. Currently, there is only limited evidence linking biofilm formation by the human pathogen *M. tuberculosis* with pathogenesis [117], although biofilm formation is speculated to enhance extracellular survival and antibiotic resistance, especially within the inflammation-driven granuloma [118]. Investigations into the matrix composition of *M. tuberculosis* pellicles predominately identified free mycolic acids [119]. This is perhaps unsurprising as mycobacteria are characterized by a three-layer cell envelope; peptidoglycan is covalently linked to the inner plasma membrane and the outer arabinogalactan and mycolic acid, and surrounded by a free-form capsule [120]. It has been reported that *M. tuberculosis* forms curli-like *M. tuberculosis p*ili (MTP) that also contribute to the biofilm matrix. MTP are insoluble, being found in the chloroform–methanol fraction during purification, with the monomer not detected using SDS-PAGE [61]. Positive Congo red binding to MTP pili was also reported [61]. *In vivo* α-MTP antibodies can be identified in sera collected from patients infected with *M. tuberculosis* [61], and deletion of *mtp* was reported to impact negatively on pellicle formation and biomass *in vitro* [63]. Although MTP insolubility and dye binding are compatible with the hypothesis of an amyloid-like fiber, further studies showed that the binding of Congo red to *M. tuberculosis* biofilms occurred in the absence of the *mtp* gene, and when screened by TEM, it was highlighted that there were not always obvious phenotypic differences between the wild-type and *mtp* mutants [62]. It is possible that some of the fibrous morphology initially identified [61] could be due to the capsule and/or matrix mycolic acids collapsing into rope-like bundles [121]. During *in vivo* infection models, the *mtp* mutant was neither sensitized to antibiotics nor attenuated for extracellular survival [62]. Thus, whether MTP are required for biofilm formation, and if they are amyloid-like fibers in form, requires further investigation.

## Protease-Sensitive Fibers in *Xanthomonas* Species

*Xanthomonas axonopodis* pv. *citri* (hereafter *X. citri*) is a phytopathogen responsible for citrus canker [122], large necrotic lesions that develop on the leaves and fruits of citrus trees. This bacterium can develop structured biofilms *in vitro* and on the leaf surface [122] and switches from an epiphytic to a pathogenic lifestyle in a process dependent on the size of the colony on the plant surface [123]. Biofilm formation of *X. citri* is reliant on an active type III-secretion system [124], which injects effector proteins into the plant host. Among these proteins are the harpins, a heat stable family of proteins encoded by the hypersensitivity-response-and-pathogenicity (*hrp*) locus [73]. When introduced to the plant cytoplasm, harpins generally initiate an immune defence known as the hypersensitivity response (HR) that is similar to programmed cell death and designed to prevent further spread of the pathogen [73]. HpaG is a three-domain harpin protein with two α-helical domains linked by a putative prion domain that was identified *in silico* [74]. Recombinant poly-histidine tagged HpaG is biologically functional, in that it induces HR in non-host tobacco leaves, and when freshly purified, size exclusion chromatography indicates that the protein forms a tetramer. When this fraction is collected, TEM reveals ring-shaped structures and the CD spectrum is predominately α-helical [74]. Over the course of 10 days, the purified protein formed an obvious gel and TEM analysis shows the formation of fibers, 5–7 nm wide, with a 220-nm minima observed by CD [74]. These fibers bound Congo red but were sensitive to proteinase-K degradation, indicating that they are not amyloid-like [74], and although It was speculated that this capacity to form fibrous aggregates may be linked to HR induction, further investigation is required.

## SDS-Sensitive Recombinant Fibers of *S. aureus* SuhB

The fibers observed to form during recombinant over-expression of the poly-histidine tagged *S. aureus suhB* gene (SaSuhB) in *E. coli* are large. With a 4-mm length, the macroscale assemblies are visible to the naked eye and can be separated from the cell culture with a 0.22-μM cheesecloth. The recombinant *E. coli* strain displays a red phenotype when grown on agar supplemented with Congo red, a phenotype that is not seen either when the empty vector or when the *E. coli* native *suhB* gene is used [120]. The authors further speculated that the protein is secreted by the curli-specific secretion system.

The recombinant fibers are sensitive to SDS degradation and appear to be extremely sticky, with *E. coli* cells remaining on the TEM grid even after extensive washing. When the polyhistidine-tagged SaSuhB is purified from crude cell lysate by Nickel-NTA affinity and subjected to size exclusion chromatography, there is a void volume peak that has been attributed to protofilaments and a peak corresponding to dimeric protein [70]. When the CD spectra of both species are compared, the protofilaments and dimers have mixed α-helical and random coil structural elements, with some β-sheet enrichment upon polymerization but no major conformational changes [70]. The secondary structure arrangement by X-ray diffraction pattern of the fibers exhibits three strong bands at 3.8, 4.7 and 10–11 Å [70]. In light of the solubility of the fibers, it seems unlikely that the protofilaments have a cross-β structure, and currently, there are no *in vivo* data available for the functionality.

## Bap Fibers by *S. aureus*

The gene encoding the biofilm-associated protein (Bap) in *S. aureus* was first identified in a transposon mutagenesis screen looking for biofilm-attenuated mutants [125]. Bap was subsequently demonstrated to play a role in the colonization of surfaces *in vitro* and in an *in vivo* infection model [125]. The cell wall fraction of *S. aureus*, grown in the presence of glucose, was shown to contain SDS-insoluble aggregates which reacted with α-Bap antibodies [71]. Bap was categorized as an amyloid-like fiber based on the binding of Congo red and ThT dyes by a synthetic internal peptide of the protein (named rBap_B). Consistent with this designation, fibrous aggregates were observed by TEM, although these experiments were performed at a low pH. As S. aureus is capable of glucose fermentation, the authors suggested the accumulation of acidic by-products may lower the pH in a biologically relevant fashion. The rBap_B peptide was found to constitute the minimal functional unit that is capable of inducing cell clumping in a Δbap mutant at pH 4.5, with faint, needle-like fibers being visible in the extracellular space by TEM [71]. The role of Bap *in vivo* needs further study; for example, there was no difference in the adhesion capacity of wild-type and Δbap strains in an implanted catheter mouse model at 4 days but the absence of bap did reduce persistence over 10 days [71]. Further structural characterization is necessary to determine if these fibers contain the cross-β hallmark structure, and whether the low pH form is of biological relevance in clinical settings.

## The Surface Adhesins of *Streptococcus mutans*

*S. mutans* has historically been considered responsible for dental caries, although recent data examining the oral microbiome have raised doubts of any single causative organism [126]. Within the polymicrobial oral biofilm, *S. mutans* is capable of producing an extracellular polysaccharide called glucan by metabolising sucrose, and utilizing it to attach to tooth enamel and other microorganisms [127], [128]. *S. mutans* also has cell surface expressed proteins that can mediate adhesion. The best-studied antigen is the P1 (or AgI/II) antigen, which binds to the glycoprotein salivary agglutinin secreted by the salivary gland [129]. Initial TEM imaging of immunogold-labeled α-P1 with *S. mutans* cells revealed a fibrillar “fuzzy” layer [64]. Subsequent characterization reveals a complex multidomain tertiary structure capable of aggregation [129], [130]. The crystal structure of the three N-terminal domains reveals an elongated 50-nm stalk on which a hypervariable head is extended with a C-terminal region anchoring to the cell [129], [130]. The *S. mutans* surface-expressed antigen P1, the antigen precursor protein WapA and the secreted SMC_63c protein have all been reported as forming amyloid fibers [48], [65]; however, their designation as amyloid fibers relies heavily on Congo red and ThT binding. *In vitro* analysis of recombinant P1 showed positive Congo red and ThT binding after a period of agitation [48], but positive Congo red binding is also shown for three wild-type strains isolated from dental caries as well as in a P1 deletion mutant [48]. This is perhaps unsurprising considering the dyes' non-specific binding to other polymers [131]. The P1, WapA and SMC_63c proteins are all sensitive to SDS depolymerization [48], [65], and no molecular structural information has been collected to suggest formation of the cross-β amyloid structure. CD spectra of the P1 C-terminal domain indicates it is predominately β-sheet [65], and SMC_63c undergoes a conformational change when exposed to pH 4 from an α-helical to predominately β-sheet composition [65]. As discussed previously, the conformational changes induced at this pH may not be biologically relevant particularly if low pH exposes regions of the protein that initiate misfolding into an amyloid cross-β architecture, although in the case of P1, WapA and SMC_63c proteins, there is currently no evidence of such tertiary structures.

## Concluding Remarks

Since the identification of curli as amyloid-like fibers present in the biofilm matrix [49], it became common to compare proteinaceous fibers from other single species biofilms to the curli. However, as highlighted here, the biophysical characterization of a cross-β structure has not been directly linked to biological activity for many of the examples in the literature (Table 1). Moreover, secondary structure data (e.g., CD spectra) do not provide evidence of a cross-β arrangement, which requires either X-ray fiber diffraction performed on aligned fibrils or atomistic methods such as solid-state NMR. Furthermore, some of the fibers discussed here are unstable when exposed to SDS and/or proteinase-K [59], [61], [65], [70], [72], [74], which is suggestive of a non-amyloid fiber structure. Finally, if additional variables such as extremely low pH or high temperatures are introduced, these can induce misfolding into an amyloid-like fibril form, which may or may not have relevance in the native biofilm. Thus, an evaluation of structural evidence available for the fiber-forming proteins present in single species bacterial biofilms reveals that currently very few meet the criteria of possessing the cross-β hallmark structure of amyloid fibers. In the absence of this, it appears logical to conclude that many of the functional fibers found in the biofilm matrix adopt forms that may not be amyloid in nature. It is important to note that this does not alter their importance to biofilm formation or the interest in understanding the mode of formation. It simply highlights the great variety of mechanisms bacteria have evolved to form protein fibers to support life in a biofilm.
